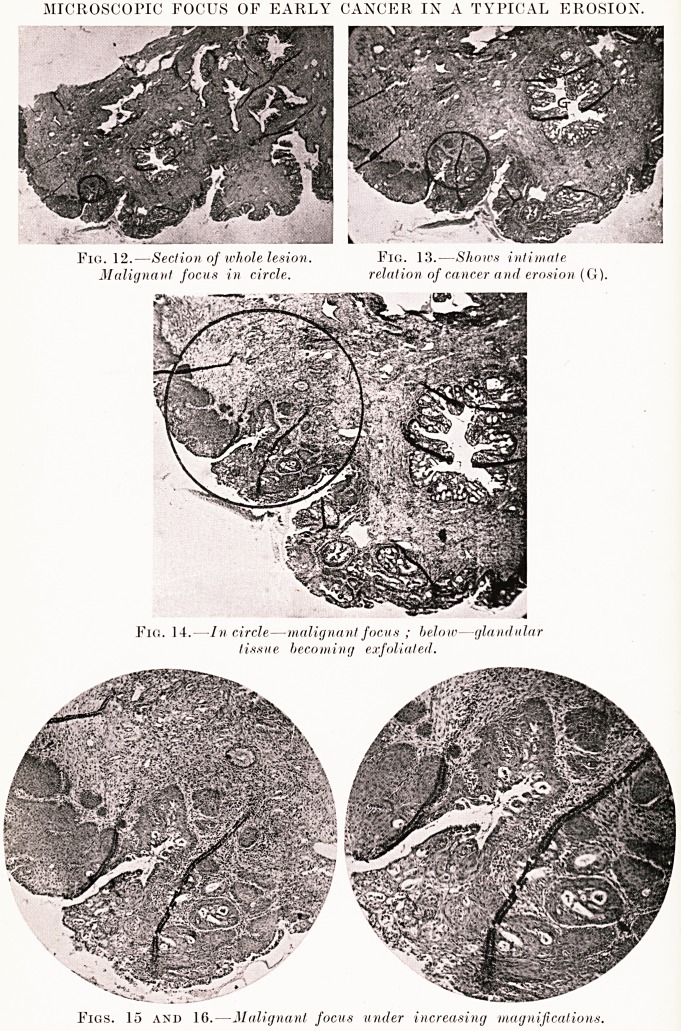# The Pathology of Cervical Erosion
1A Paper read at the Bath and Bristol Branch of the British Medical Association, May 26th, 1926.


**Published:** 1926

**Authors:** Geoffrey Hadfield

**Affiliations:** Pathologist to the Bristol General Hospital


### THE PATHOLOGY OF CERVICAL EROSION.[1]

BY

GEOFFREY HADFIELD, M.D., M.R.C.P. Lond.,

*Pathologist to the Bristol General Hospital.*

---

THE simple epithelium of the cervix uteri begins to differentiate about the third month of fœtal life into two types, that lining the cervical canal becoming columnar and, by invagination, forming a system of mucus-secreting glands, that covering the vaginal portion becoming squamous and laminated. At birth, the boundary between these two epithelia normally lies just within the lips of the external os, so that no glandular tissue is visible from the vagina. Occasionally, by faulty development, the columnar epithelium of the canal covers the vaginal portion of the external os over a circular area of varying size, producing the so-called congenital erosion of infants. This condition, purely developmental in origin, is a congenital, or, strictly speaking, a dysembryoplastic heterotopia and is usually corrected, the exposed ring of gland tissue being exfoliated by inward growth of the squamous tissue as far as the boundary-line. During this process and during the epithelial differentiation of the normally-developed cervix the two types of epithelium are in close association, and it is usually agreed that they replace each other with comparative ease and that degradation of columnar to squamous cells occurs readily.

The familiar acquired erosion of multiparous women,

[1] A Paper read at the Bath and Bristol Branch of the British Medical Association, May 26th, 1926.

although in all probability of different origin, is nevertheless due to heterotopia of cervical gland tissue. The epithelium of the external os disappears and proliferating gland tissue continuous with that lining the canal replaces its squamous lining. It is possible that in a few cases the condition is explained by inflammatory hyperplasia of congenitally heterotopic glandular tissue; some cases may be due to actual ectropion of the lining of the canal during labour, but it appears that the majority are due to the chronic endo-cervicitis of repeated pregnancies or of gonococcal infection, and that the squamous lining of the os is actually pierced by inflamed glandular tissue which presents at the external os as a reddened, roughened and irregularly shaped area—the simple glandular erosion (see Figs. 2 and 3). Several factors tend to endow the common glandular erosion with a considerable degree of chronicity and to alter its structure. In the first place, surface infection is common, so that increased vascularity and replacement fibrosis are present in inverse ratio. Next, the glandular tissue undergoes secondary changes, frequently becoming cystic (Fig. 3), and when of long standing the surface of the erosion often becomes covered by fine delicate papillæ (papillary erosion) due to projection as multiple polypi of the inflamed œdematous interstitial tissue lying between the gland mouths (Figs. 5 to 8). Finally, there is always a strong tendency for the squamous epithelium at the margin of the lesion to grow inwards and cover it, and for islands of surviving squamous cells isolated in it to grow over it. If this is successful, sheets of squamous cells grow into the ducts and tubules of the glands, which become exfoliated over large areas. In many cases this process of epidermisation is considerably delayed by the chronic irritation of surface

### PLATE VI.

FIG. 1.—*Diagram illustrating varieties of erosion and changes leading to malignant disease.*

FIG. 2.—*Simple glandular erosion in early stage.*

FIF. 3.—*Preceding under low power.*
S.—Infected surface.

FIG. 4.—*Cystic erosion. Low power.*

### PLATE VII.
### PAPILLARY EROSION.

FIG. 5.—*Natural size.*

FIG. 6.—*Edge of lesion* ( ×8).

FIG. 7.

*Surface at external os* ( ×8).

FIG. 8.
*Section of preceding, low power.*

and endocervical infection, whilst in some the repeatedly frustrated attempts at healing end in the production of foci of atypical and irregularly-laminated squamous-celled overgrowth, leading to the formation of islands of leukoplakia, to wart-like local overgrowth or to uniform coarse papilloma formation.

Along with these changes of gross structure there occur all those changes in the cells themselves which are styled pre-cancerous, including cellular and nuclear heteromorphism, increased mitoses and nuclear hyper-chromatism. Such appearances are a commonplace in the routine examination of cervical tissue removed for intractable erosion, and are precisely the histological changes which so frequently precede malignant disease on every squamous-lined surface in the body. It cannot be too strongly urged, however, that such changes are not sufficient for a diagnosis of malignant disease until infiltration of deep tissue becomes manifest, and there is no doubt, as in the case of similar lesions of the tongue, that this dangerous-looking but non-infiltrating epithelial overgrowth may exist in an unhealed lesion for so long a time, that its malignant potentialities become insignificant, whilst in a few cases satisfactory healing may follow. In other words, although the malignant potentialities of these lesions are high, one cannot, with certainty, predict a malignant termination for them. Herein lies the immense importance of deciding how frequently intractable erosion is the forerunner of cervical cancer. It is not enough to find the two lesions clinically associated : they are both so common that a very large series of clinical records extending over many years would be necessary to gain reliable information. The problem is precisely similar to the determination of the frequency of malignant transformation of a chronic gastric ulcer, except that in the case of erosion the histological criteria necessary for proof are considerably less liable to lead one into fallacy than in the case of ulcer-cancer of the stomach.

To establish the fact that an erosion has become cancerous we must find in the same cervix the typical glandular heterotopia of erosion in intimate relation with the infiltration of cancer, and satisfy ourselves that the erosion was the primary lesion. We must also find out how far the secondary changes produced by cancer in the glandular tissue of the cervix may copy those of erosion, and how far those produced by erosion may copy those of cancer.

I have for some time past examined all types of erosion coming to the laboratory as routine specimens ; and, employing these criteria, I find that when any case has shown clinical signs of malignant disease, it is usually difficult to demonstrate the typical lesion of erosion in intimate relation with the new growth, as the latter very rapidly overwhelms what is, after all, a superficial process. The specimens shown in Figs. 9 and 10 are, however, exceptions to this, erosion and cancer existing side by side. From the material I have seen the most conclusive evidence comes from cases in which a chronic and intractable erosion was excised, but in which clinical evidence of malignant disease was lacking. In such cases, if many sections are cut, it is not at all uncommon to find single and often microscopic foci where the widespread squamous overgrowth has assumed the characters of malignant disease.

The earliest case I have seen is that illustrated in Figs. 12 to 16, from which the minute dimensions of the focus can be judged by following it through increasing magnifications. It is in such material, in which cancer is not clinically obvious nor even suspected, that I have

### PLATE VIII.

Fig. 9.—*Malignant transformation of erosion.*
Heaped-up malignant tissue replaces cervix except at E and E1, where appearances—confirmed by section—are those of erosion.

Fig. 10.—*Half a malignant cervix.*
Typical lesion of erosion remains over area E.

Fig. 11.—*Carcinomatous ulceration.*
No trace of erosion microscopically. History suggests previous erosion.

### PLATE IX.

### MICROSCOPIC FOCUS OF EARLY CANCER IN A TYPICAL EROSION.

FIG. 12.—*Section of whole lesion. Malignant focus in circle.*

FIG. 13.—*Shows intimate relation of cancer and erosion* (G).

FIG. 14.—*In circle—malignant focus ; below—glandular tissue becoming exfoliated.*

FIGS. 15 AND 16.—*Malignant focus under increasing magnifications.*

been able to satisfy myself that a considerable percentage of cervical cancers probably arise during the unsuccessful healing of the chronic types of cervical erosion. As the malignant change arises during the healing process when this is repeatedly frustrated by chronic irritation, there can be little doubt that by applying strong chemical irritants to erosions which are still unhealed after a period of years we copy very exactly the conditions under which cancer has been produced experimentally in animals.

Thus, when an erosion has reached such a stage of unhealed chronicity that atypical squamous overgrowth is likely to be present, local irritants should be avoided and wide excision practised.

## Figures and Tables

**Fig. 1. f1:**
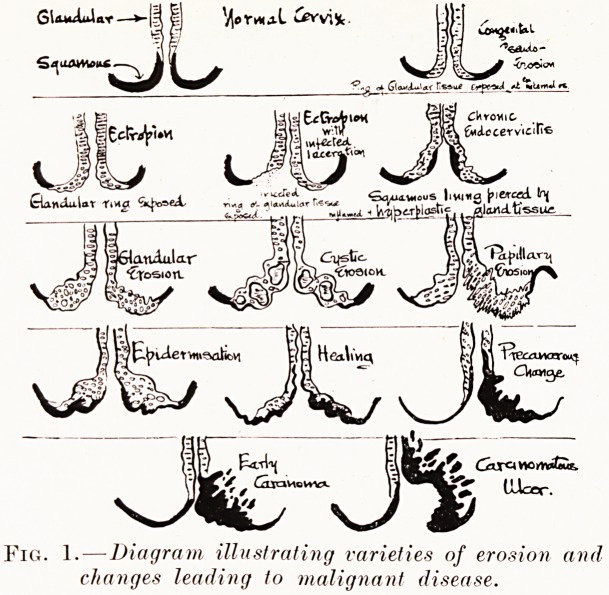


**Fig. 2. f2:**
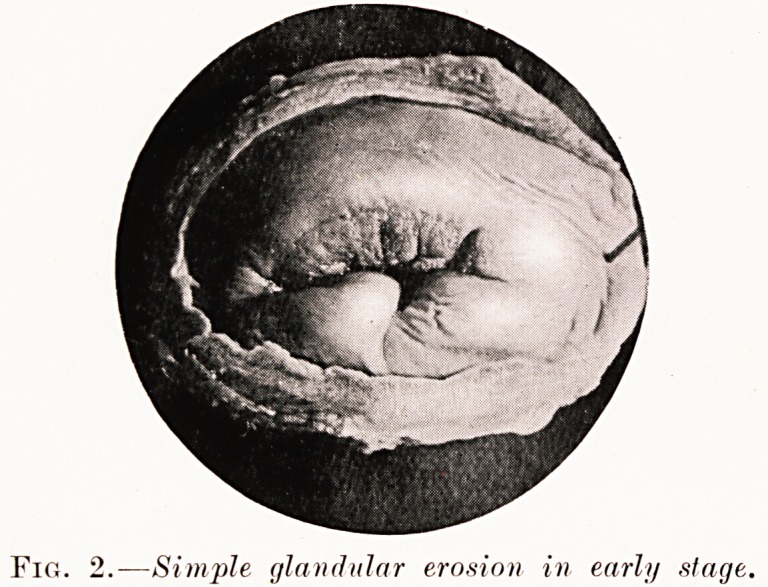


**Fif. 3. f3:**
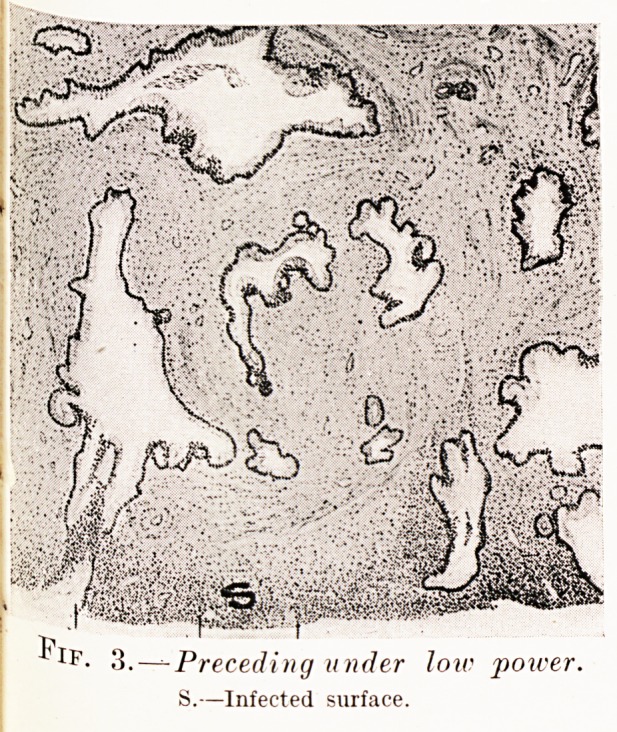


**Fig. 4. f4:**
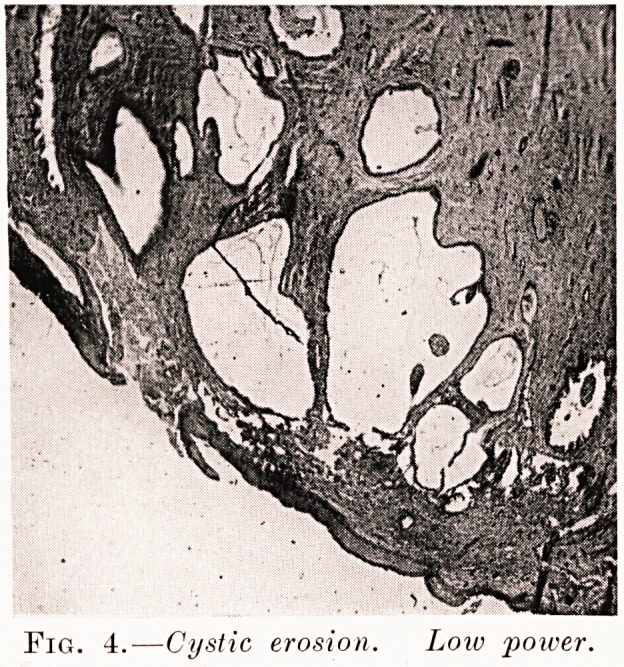


**Fig. 5. Fig. 6. Fig. 7. Fig. 8. f5:**
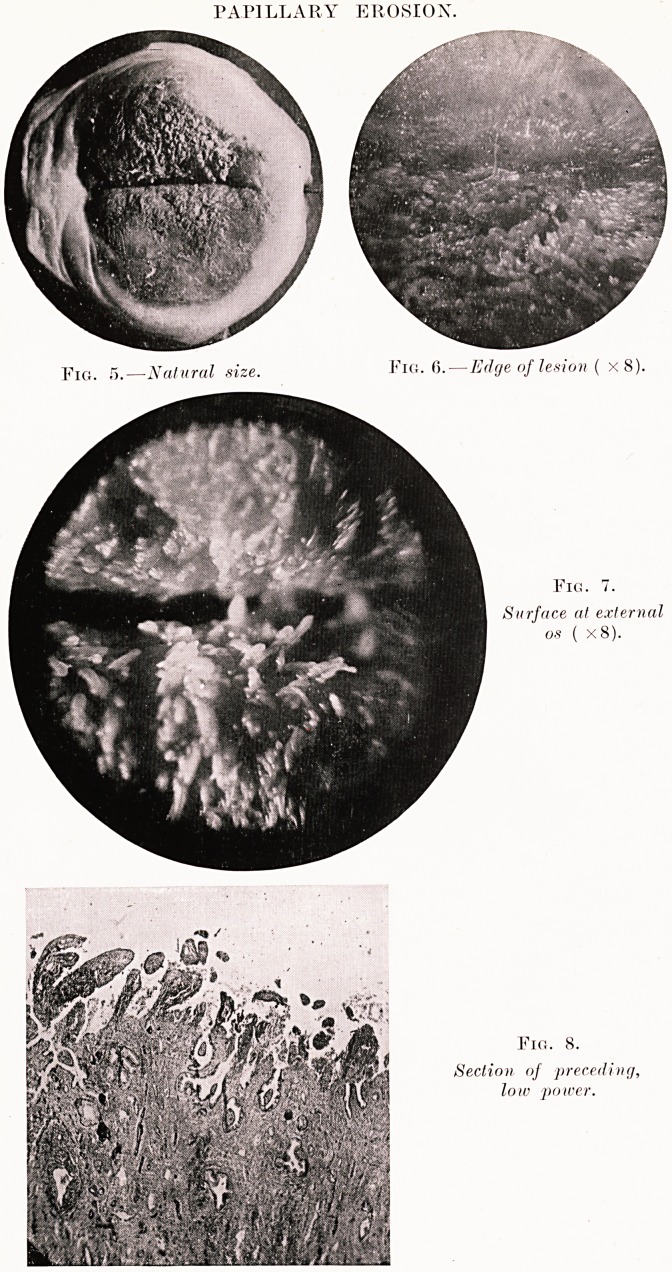


**Fig. 9. f6:**
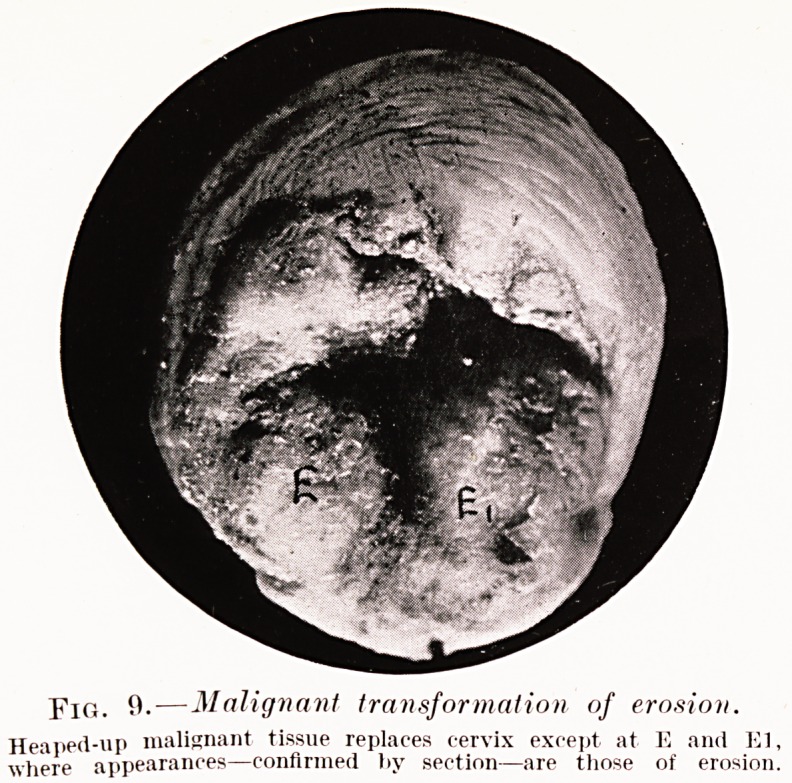


**Fig. 10. f7:**
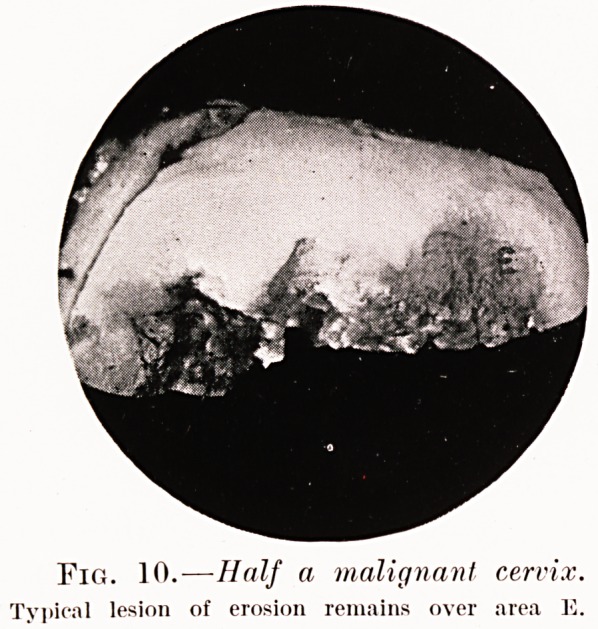


**Fig. 11. f8:**
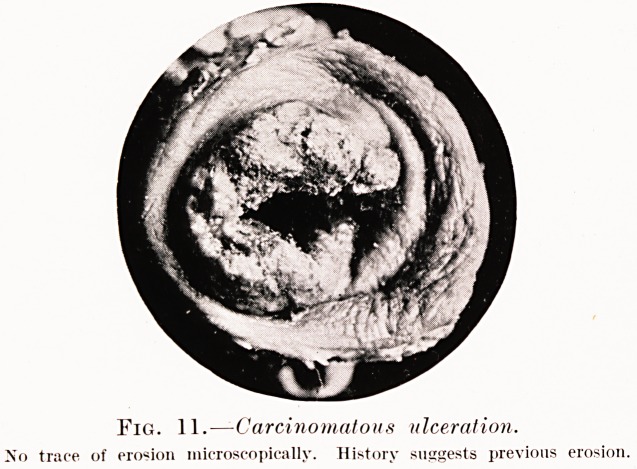


**Fig. 12. Fig. 13. Fig. 14. Figs. 15 and 16. f9:**